# Editorial: Soundscape Assessment

**DOI:** 10.3389/fpsyg.2019.02514

**Published:** 2019-11-08

**Authors:** Östen Axelsson, Catherine Guastavino, Sarah R. Payne

**Affiliations:** ^1^Gösta Ekman Laboratory, Department of Psychology, Stockholm University, Stockholm, Sweden; ^2^School of Information Studies and Centre for Interdisciplinary Research in Music Media and Technology, McGill University, Montreal, QC, Canada; ^3^The Urban Institute, Heriot-Watt University, Edinburgh, United Kingdom

**Keywords:** soundscape, standardization, assessment, methodology, perception

## Soundscape Research, 50 Years

In 2019, soundscape research celebrates 50 years as a scientific field. In 1969, the first scientific article using the term “soundscape” was published in the inaugurate issue of the premier scientific journal for environmental psychology, Environment and Behavior (Southworth, 1969). The author was Michael Southworth, a PhD student in city planning at MIT in Boston, today Professor Emeritus of Urban Design at UC-Berkley. The article was based on his Master's Thesis in city planning, which he completed at MIT in 1967.

In the 1970s and 1980s, soundscape was largely associated with the Canadian composer R. Murray Schafer at Simon Fraser University in Vancouver. In 1972, Schafer begun the World Soundscape Project by a detailed study of the soundscape of Vancouver.

The topic gained international momentum after being introduced to the wider community of noise and health researchers at the International Congresses on Acoustics in Seattle 1998. In 2008, the International Organization for Standardization (ISO) formed a working group on the topic to develop the ISO 12913 series, which is the first international standard in this field.

Part 1 of ISO 12913 was published in August 2014. It defined the term of “soundscape” as “acoustic environment as perceived or experienced and/or understood by a person or people, in context” (ISO, 2014). It also provides a conceptual framework, distinguishing the acoustic environment, as a physical phenomenon, from the soundscape, as a perceptual construct. Part 2 identifies data collection and reporting requirements (ISO, 2018), while Part 3 will identify data analysis aspects. The present Research Topic was initiated to support the development of the ISO 12913 series by investigating methods for soundscape assessment.

## Diversity of Approaches

A wide range of methods and subjects are covered in this Research Topic, indicating that soundscape assessment should be approached from a holistic, multisensory perspective to capture outcomes that extend well-beyond auditory judgments. Contributions encompass theoretical and practical approaches, highlighting their complimentary, and informative role in furthering soundscape assessments.

Two contributions investigated the relationship between public space usage and soundscape. Bild et al. used behavioral mapping and questionnaires to investigate how social interaction influences soundscape assessment. Meng et al. investigated how music in a public space influences crowd density and walking patterns. In these instances, behavioral observations identified variations in soundscape assessments by groups of people.

Two contributions took a cognitive approach by using free sorting tasks conducted by individual participants. Bones et al. developed a sound taxonomy to investigate how people categorize environmental sounds. Aletta et al. identified holistic acoustic properties through sorting of spectrograms, instead of soundscape recordings.

Several contributions focus on refining or improving existing soundscape assessment methods, particularly semantic scales that are often used in questionnaire studies. Welch et al. developed semantic scales based on creative writing as a way of exploring a wider range of soundscape descriptors. Payne and Guastavino investigated the validity of the Perceived Restorativeness Soundscape Scale (PRSS) using psycholinguistic analysis. van den Bosch et al. developed a theoretic framework to provide the underpinnings of existing results in soundscape research and a rational for current assessment models.

Contributions also highlight the importance of person-related and contextual factors in soundscape assessment, which have previously received limited attention. Sun et al. investigated how individual differences in the ability to process audio-visual information (named *audio-visual aptitude*) influences the interaction between landscape and soundscape appraisal. Benfield et al. studied how attitudes to motorized recreation in national parks and to its regulation may moderate the effect of the sound of motorized recreation on scenic evaluation. Memoli et al. discovered that deviation from the expected flight path influences noise annoyance from incoming, landing aircrafts.

## Collaboration Built on Diversity

The diversity of methods among the contributions reflects the two-way interaction between theory and practice, revisiting the traditional dichotomy between deductive and inductive approaches. While theories may guide concrete soundscape interventions, the complexity of real-world applications inform and enrich theories and models. The methods include field and laboratory studies, as well as qualitative and quantitative methods, suggesting that no single method may capture all the different facets of a soundscape.

With this diversity of soundscape assessment methods, integration of the various approaches, and comparability across results is increasingly difficult. Soundscape researchers consider different approaches depending on the object of study (e.g., individual sensory experience, group behavior, acoustic properties, or invested meaning). The diversity of research methods used suggests that soundscape, both as an object of study and as a field of research, is still under development. Consequently, standardization efforts should focus on identifying or developing a reference method for enhanced comparability among studies, as opposed to a single soundscape method. This observation reflects the outcome of ISO/TS 12913-2 that recommends multiple assessment methods (ISO, 2018), as a single method could not be agreed upon.

The increasing interest in contextual and person-related factors is worth noting, and reflects the ISO definition (ISO, 2014). Contextual and person-related factors extend beyond auditory judgment, providing a more holistic representation of the soundscape. A focus on context also enables the inclusion of applied research, around practical soundscape interventions, alongside more fundamental research that advances theory development.

Another recurring challenge in soundscape research is the main sources of variance: individual variation among participants providing the soundscape assessments, and variation among soundscapes. Essentially, soundscapes result from a variety of sound sources, in varied contexts. Frequently, researchers investigate only one kind of place, such as parks or plazas. This provides an in-depth understanding of issues related to these particular contexts, but limits the generalizability to other places, where sound sources and contextual factors will vary. Recognizing the range of contexts and investigating the transferability of research findings from one context to another provides further directions for soundscape assessment research.

While it is important to relate critically to existing methods and results, and to examine their validity, it is also important to seek a common ground and common objectives for a research field to develop further. Consequently, it is important to encourage continued and deepened international and interdisciplinary collaboration.

## Author Contributions

All authors listed have made a substantial, direct and intellectual contribution to the work, and approved it for publication.

### Conflict of Interest

The authors declare that the research was conducted in the absence of any commercial or financial relationships that could be construed as a potential conflict of interest.
